# Predictors of clinically important improvements in occupational and quality of life outcomes among mental health service users after completion and follow-up of a lifestyle intervention: multiple regression modelling based on longitudinal data

**DOI:** 10.1186/s40359-019-0359-z

**Published:** 2019-12-17

**Authors:** Jenny Hultqvist, Kristine Lund, Elisabeth Argentzell, Mona Eklund

**Affiliations:** Department of Health Sciences, Mental Health, Activity and Participation, Lund University, Lund, Sweden

**Keywords:** Occupational therapy, Psychiatric rehabilitation, Occupational balance, Occupational engagement, Quality of life, Mental illness

## Abstract

**Background:**

Balancing Everyday Life (BEL) is a new activity-based lifestyle intervention for mental health service users. An earlier study found BEL to be effective in increasing occupational engagement, occupational balance, activity level, and quality of life scores when compared with a care-as-usual group. However, it is unclear whether care context and socio-demographic, clinical and self-related factors at baseline also influence the results. Thus, the aim of the current study was to explore whether such factors could predict clinically important improvements in occupational and quality of life aspects.

**Methods:**

Participants were interviewed and filled out self-report questionnaires before starting the 16-week intervention (*n* = 133), upon completion (*n* = 100), and 6 months following (*n* = 89). Bi-variate and multi-variate statistical analyses were performed.

**Results:**

Several baseline factors were associated with clinically important improvements, but few predictors were found in the multivariate analyses. Having children was found to be a predictor of improvement in occupational engagement at BEL completion, but reduced the chance of belonging to the group with clinically important improvement in activity level at follow-up. Regarding occupational balance, having a close friend predicted belonging to the group with clinically important improvement in the leisure domain. At BEL completion, other predictors for improvements were female gender for the self-care domain, and self-esteem for the home chores domain. At follow-up, psychosocial functioning and lower education level predicted general balance. None of the factors explored in this study were found to be predictors for improvements in quality of life.

**Conclusions:**

Few of the studied care context, socio-demographic, clinical and self-related factors were found to predict clinically important improvements in occupational engagement, activity level, occupational balance, or QOL. This study, together with previous studies showing positive results, suggests that BEL can be an appropriate intervention in both community and clinical settings, and can support improvement in occupational aspects and QOL for participants with diverse socio-demographic, clinical, and self-related characteristics.

**Trial registration:**

This study is part of a larger research project that is registered at ClinicalTrials.gov. Reg. No. NCT02619318.

## Background

Occupational therapy suggests that engagement in everyday occupations affects health and quality of life (QOL) [1, 2]. All too often, individuals with mental illness lack opportunities for engagement in meaningful everyday occupations and experience deteriorated QOL [3, 4]. This is detrimental to personal recovery [5], a concept which can be described as a unique process of improved mental health and well-being and creating hope, meaning and purpose in life despite of illness or symptoms [6–8]. Considering a person’s occupational circumstances such as occupational engagement, activity level and occupational balance, as well as QOL, is therefore of importance for recovery-oriented practice [5, 9].

Following that line of research findings, the Balancing Everyday Life (BEL) was developed with the aim of supporting mental health service users to make personally meaningful changes toward attaining a better balance in daily life, and improving QOL [10]. BEL is an occupational therapy intervention developed for people using community-based and specialized outpatient psychiatric services. BEL is founded on research on occupational engagement, meaning, and balance among mental health service users [11, 12], lifestyle interventions focusing on patterns of daily occupations [13, 14] as well as principles from personal recovery-oriented practice, such as personalized short-term goals [8].

The BEL intervention was organized as a 12-week-long group-based course with two additional booster sessions in weeks 14 and 16. Groups were generally composed of four to six participants. A course manual provides guidance and materials for group leaders regarding the weekly topics and related exercises. A corresponding workbook binder exists for the participants. Together with the group, participants reflect on their past and present occupational patterns and discuss the presented topics in order to explore and develop a personalized balance in daily life within areas such as social relationships, productive occupations, meaningful leisure time, physical exercise, rest and relaxation, as well as health-promoting approaches to nutrition and sleep [15]. Participants set personally motivated goals, based on their unique needs and desire for change, and work on their goals as home assignments in a real-life context. This could include identifying and engaging in meaningful occupations and relationships, setting personal goals related to diet and nutrition, and working with one’s daily rhythm and routines [10]. Two mental health professionals led the BEL intervention, at least one of which was an occupational therapist. In settings with only one occupational therapist, the co-leaders were, for example, nurses or social workers. All the occupational therapists who were group leaders had undergone a specifically developed two-day education.

A qualitative study [16] found that the BEL participants and group leaders experienced the intervention as positive and appreciated the structure and content of the intervention, yet desired more sessions. A RCT [10] evaluating the effectiveness of BEL showed that the BEL group (*n* = 100) improved more than the control group (*n* = 80) from baseline to 16 weeks on the occupational aspects occupational engagement (*p* < 0.001), activity level (*p* = 0.036), and occupational balance (*p* = 0.042). Other outcomes were reduced symptom severity (*p* = < 0.046) and improved level of psychosocial functioning (*p* = 0.018). The group differences on occupational engagement and activity level remained at the six-month follow-up, when the BEL group (*n* = 89) had also significantly improved their QOL (*p* = 0.006). It is not known, however, whether the RCT outcomes are influenced by the care context or other potentially instrumental factors, such as socio-demographic and clinical factors and self-concept in terms of self-esteem and self-mastery, which was the rationale for the present study.

As described above, the participants in the BEL intervention group showed improvements in QOL and several aspects of occupation; occupational engagement, activity level and occupational balance. Occupational engagement concerns the actual doing while also stressing the individual’s experience, purpose, and sense of meaning in the occupation [17, 18]. Research indicates that clinical and sociodemographic factors and self-factors may influence the ability to engage in everyday occupations. Bejerholm and Eklund [11] found that a higher level of occupational engagement was associated with fewer psychiatric symptoms and that gender was associated with the type of occupations people engaged in. The results of two other studies found that a higher level of occupational engagement among mental health service users was associated with having a close friend, a higher educational level, being employed or studying [19], and better self-mastery [20].

Occupational engagement is linked to other occupational aspects such as activity level and occupational balance. Activity level denotes the number of activities that an individual performs on an everyday basis [21]. The results of a study by Eklund and Leufstadius [22] showed that a higher activity level related to less severe psychiatric symptoms, better psychosocial functioning, and better self-mastery.

While activity level is closely related to occupational balance, the latter is defined as the individual’s perception of having the right amount of and variation in occupations [23]. Occupational balance is affected by our patterns of everyday occupations [24], and research shows that people with mental illness are at risk for occupational imbalance, which can include patterns of having very few or too many occupations, or a variation between both [12, 25]. A study among people with schizophrenia reported an association between being under-occupied and having negative symptoms [26]. Another study showed that a risk factor for under-occupation in work activities was younger age, and a risk factor for overall imbalance was a higher educational level [27]. The latter study also showed that better self-esteem and self-mastery were associated with better occupational balance.

There is also research indicating that clinical, sociodemographic and self-factors may influence QOL in people with mental illness. Better QOL was found to be associated with younger age and fewer psychotic symptoms [28], less severe depressive and anxiety symptoms [29, 30], better psychosocial functioning [31], a higher educational level [32], better self-esteem [33], and better self-mastery [34].

Much of the existing research is cross-sectional and research is lacking on potential predictors of change for people with mental illness attending an activity-based intervention. It was therefore felt warranted to perform an exploratory study on the BEL occupational and QOL outcome variables, while also investigating the importance of potential predictors; specifically care context, sociodemographic and clinical factors, and self-factors as predictors. The purpose was to deepen our knowledge of how these factors influence occupational aspects and QOL, and whether they may play a role in the possibility for benefitting from the BEL intervention.

## Methods

This longitudinal study was part of a larger RCT project based on cluster randomization, evaluating the effectiveness of the BEL program [10], and adheres to the CONSORT guidelines. The control group received standard mental health occupational therapy. The present study’s focus was exclusively on the participants who had been randomized to receive the BEL intervention. They filled out self-report questionnaires targeting aspects of occupation and well-being on three occasions – at baseline, after completed intervention (at 16 weeks) and at a six-month follow-up. On these occasions, a research assistant rated the participants’ symptom severity and level of functioning.

The aim of the current study was to explore which baseline factors could predict clinically important improvements in occupational engagement, activity level, occupational balance, and QOL among mental health service users at BEL completion and follow-up. Factors to be considered as potential predictors were care context, socio-demographic and clinical factors, and self-factors.

### Selection of settings and participants

Settings invited to enter the larger project provided outpatient specialized psychiatry (general psychiatry and psychosis care) and community-based psychiatry care (activity-based day centers) in three regions in the south and west of Sweden. Recruitment criteria for the settings included not currently being involved in another research project, not undergoing a re-organization, and having at least one occupational therapist employed at the setting [10]. In settings where staff agreed to participate, a gatekeeper (an on-site occupational therapist) identified clients according to the inclusion criteria: a) having a self-reported occupational imbalance (assessed in an interview with the occupational therapist), b) age of 18–65 years, c) the main diagnosis not being substance abuse, d) no comorbidity of dementia or intellectual disability and e) sufficient literacy in Swedish to participate in the data collection.

**Fig. 1:**
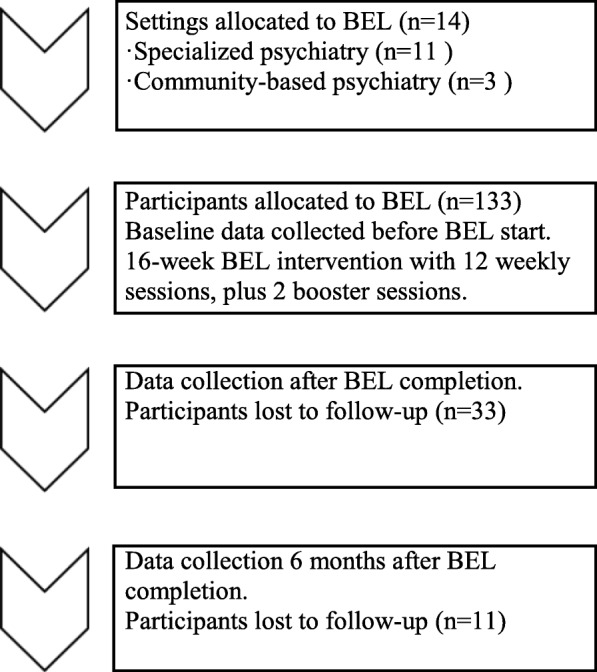
An overview of settings, participants, and quantitative data collection points in the BEL research project

The BEL intervention group included 133 participants from 14 sites; 106 participants took part in BEL as part of specialized psychiatry services, and 27 participants attended BEL in community-based psychiatry settings (Fig. 1). The power analysis for the RCT included consideration of the effect of clusters (the different settings) and indicated that 65 participants in the BEL group and 65 in the comparison group were needed to detect the desired difference with 80% power at *p* < 0.05 [10]. This study addressing 133 BEL participants was thus well-powered.

### Data collection

Twelve research assistants who all had previous experience of working with people with mental illness performed the data collections. Eleven had an occupational therapy background and one research assistant was a final year psychology student. All the research assistants received training in using the instruments prior to contacting the participants. The data collection took place in a private room at the individual sites, took approximately 45–90 min, and participants could take breaks at any time. The choice of instruments was based on two considerations. The first was that we needed a broad battery of instruments to cover potentially important outcomes. The instruments thus had to be sufficiently broad but, secondly, not too time consuming in order to avoid stress and exhaustion among the study participants. The data was collected between November 2012 and March 2015. The recruitment ended when all eligible settings in the strategically selected regions had been invited.

#### Socio-demographic and clinical factors

Socio-demographic factors such as gender, age, civil status, living situation and educational level were collected with a questionnaire devised specifically for this study. The participants were also asked for their self-reported diagnosis/psychiatric problems. Based on these self-reports a specialist psychiatrist made ICD-10 diagnoses for use in the research data, according to a previously validated procedure [35].

#### Psychosocial functioning

Psychosocial functioning was measured by the Global Assessment of Functioning, GAF [36], which consists of separate scales for symptoms (GAF-S) and functioning (GAF-F). The scale has 100 scoring possibilities, from 1 to 100; a higher value on GAF- S indicates fewer and/or less severe psychiatric symptoms and a higher value on GAF-F indicates better psychosocial functioning. The 100 scoring possibilities are broken into ten intervals. The rater identifies the appropriate interval, then decides if the score is at the lower or the higher end of the 10-point interval, and finally chooses the exact rating. The GAF rating was performed by the research assistants who had received specific training and gone through calibration for the GAF rating. The GAF has demonstrated good inter-rater reliability after minimal training [37].

#### Self-factors

Two self-factors were addressed in this study, self-esteem and self-mastery, which are psychological resources that have shown to be associated with positive mental health and well-being outcomes [38, 39]. Two instruments were used; the Rosenberg self-esteem scale [40] and the Pearlin Mastery Scale [41]. The Rosenberg self-esteem scale covers ten different aspects of self-esteem, including feeling like a person of worth. The present study used the yes/no response format as recommended by Oliver and colleagues [42]. Scoring involves a method of combined ratings of five negative and five positive self-esteem response items. The mean scores for the positive and the negative items are calculated separately, where the possible average score that ranges between zero and one for both. Thereafter, the negative average score is subtracted from the positive average score resulting in an average self-esteem score that can vary between − 1 and 1. Psychometric testing has found the Swedish version of the Rosenberg Self-esteem scale to have good psychometric properties in terms of internal consistency, criterion, convergent and discriminant validity, and sensitivity to change [33].

The Pearlin Mastery Scale consists of seven items with four rating alternatives where four indicates the highest level of mastery, the possible sum score ranges between 7 and 28 and a higher score indicates stronger self-mastery. The questions reflect the individuals’ perceptions of control over factors that affect their lives. Rasch analysis of the Swedish version, Mastery-S, has shown acceptable reliability and good known-groups validity, and that the scale represents a logical continuum of the measured construct [43].

#### Occupational engagement

The Profiles of Occupational Engagement among people with Severe mental illness (POES) [44, 45], was used to measure occupational engagement. The POES consists of a 24-h diary that has four columns; for the activity performed, the social context, the geographical context and reflections/feelings. Based on the diary, a rating is made by a professional on nine items expressing level of occupational engagement on a four-point rating scale. The POES has shown good psychometric properties in terms of inter-rater agreement and construct validity [45, 46]. The current study was based on a self-report version of POES. The possible sum score ranges between 9 and 36, a higher score indicating more engagement.

#### Activity level and occupational balance

Activity level and occupational balance were measured by means of Satisfaction with Daily Occupations and Occupational Balance (SDO-OB) [27]. The SDO-OB addresses subjective perceptions of everyday activities within four categories – work, leisure, home chores and self-care. Each category has 3–4 items where the person first answers whether he/she currently performs the activity or not. The sum of yes-answers forms a measure of the level of activity, with a possible range between 1 and 13. After answering yes or no, the person rates his/her level of satisfaction with the activity, but the satisfaction scale was not used in this study. The activity balance questions included in SDO-OB reflect a time allocation perspective on activity balance and ask whether the individual does too little, just enough or too much within the four categories. There is also an overarching question about general activity balance. The SDO-OB balance questions are rated on a 5-point response scale from doing way too little (− 2) to doing way too much [2]. The balance items from SDO-OB has shown to have satisfactory construct validity [27].

#### Quality of life

To address QOL the Manchester Short Assessment of Quality of Life (MANSA) [47] was used. MANSA includes a subjective rating of general life satisfaction and satisfaction with 11 domains of QOL (work, financial situation, social relations, leisure, accommodation, living situation, personal safety, family relations, sexual relations, and physical and mental health). The individual rates satisfaction on a scale ranging from 1 = “could not be worse”, to 7 = “could not be better”. The mean ratings from the different domains form a general QOL score with a possible range between 11 and 77. Higher scores denote better QOL. The Swedish version of MANSA has been found to be psychometrically sound in terms of internal consistency and construct reliability [48].

### Statistical analysis

In the first part, bivariate analyses explored possible associations between the selected potential predictors and occupational and QOL aspects. Change variables based on differences in scores between completed BEL and baseline, and between follow-up and baseline, were calculated for the dependent variables occupational engagement, activity level, occupational balance domains, and QOL. These change variables were continuous and could be positive or negative, depending on the direction of the change. Associations between these targeted change variables and possible predictors in terms of care context and socio-demographic, clinical and self-related factors were analysed. The analyses performed were Spearman correlations (age, GAF symptoms, GAF functioning, self-esteem and self-mastery), the Mann-Whitney U-test (gender, civil status, has children, has a close friend, has seen a friend during the last week, care context, and diagnosis [depression vs. other]), and the Kruskal-Wallis test (living condition and education).

In the next part of the analysis, the calculated change variables were dichotomized according to a cut value (C), which was the value corresponding to a change/improvement at an effect size (ES) of 0.5, indicating a medium effect size [49], (C = ES * SD_0,_ where SD_0_ = standard deviation at baseline). An effect size of 0.5 has been suggested to be of clinical importance [50]. The terms “improvement” and “clinically important improvement” will be used interchangeably in the results and discussion to denote a positive change of that size.

A series of logistic regression analyses were then performed, one for each dependent variable, regressing the potential predictors from the bivariate analyses against the dichotomized change variables pertaining to clinically important improvements in occupational engagement, activity level, the targeted occupational balance domains and QOL. The authors used the Enter method for the predictor models, entering one independent variable at the time.

The level for a statistically significant *p*-value was set at *p* < 0.05; however, potential predictor variables showing an association at *p*-values < 0.10 with the dependent variable at target were included in the multivariate analyses. Missing data was handled by calculating the individual’s mean of the non-missing values in the instrument and then replacing the missing values with this individual mean. Imputation was only performed if at least 75% of the items were filled.

The software used was the IBM SPSS version 25. The advice of an expert statistician was sought at the design stage of the study.

## Results

Sociodemographic and clinical data of the participants are presented Table 1. As seen there, most of them were women and lived in a flat or house of their own. About one third received some type of housing support. Just below half of them had children. The majority used specialised psychiatry and the most common self-reported diagnostic group was mood and anxiety disorders.

**Table 1:** Baseline characteristics of the participants

Characteristics	*N* = 133
Women (%)	77
Age (mean, SD)	40 (11)
Married or cohabiting (%)	30
Living situation (%)	
Own house or flat, no support	66
Own house or flat, with support	25
Supported housing	2
Lodging	7
Education (%)	
Nine-year compulsory school or lower	18
Upper secondary school	59
College/university education	23
Having children (%)	47
Having a friend (%)	83
Having seen friend (%)	63
From specialized psychiatry (%)	80
Self-esteem (mean, range)	−0.2 (−1 – 1)
Self-mastery (mean, range)	17 (10–27)
GAF symptoms (GAF-S) (mean, range)	52 (27–80)
GAF functioning (GAF-F) (mean, range)	50 (30–90)
Diagnosis (%)	
Mood/anxiety disorders	52
Psychosis	19
ADHD/ADD	23
Other	6

Internal attrition in a number of subjects between 1 and 11 occurred on the variables

### Findings from descriptive and bivariate analyses

Table 2 displays descriptive statistics for the outcome variables at baseline. All mean ratings of occupational balance were on the negative side, indicating under-occupation. Theother mean ratings indicated a situation around or above the middle of the respective scales.

**Table 2:** Baseline statistics for the outcome variables occupational engagement, activity level, occupational balance and QOL

Variable (possible range)	Mean (range)	Sd
Occupational engagement (9–36)	20.4 (9.0–32.0)	4.8
Activity level (1–13)	7.37 (2–12)	2.17
Occupational balance		
Work balance (−2–2)	−0.41 (− 2 – 2)	0.87
Leisure balance (− 2–2)	− 0.63 (− 2 – 2)	0.93
Home chores balance (− 2–2)	−0.41 (− 2 – 2)	1.00
Self-care balance (− 2–2)	− 0.48 (− 2 – 2)	0.79
General balance (− 2–2)	−0.63 (− 2 – 2)	0.93
QOL (11–77)	32.1 (15.5–52.0)	8.50

Internal attrition of subjects occurred on the variables, between 3 and 7

For the variable occupational engagement the attrition was 52. Negative values on

Occupational balance indicate under-occupation

Associations between possible predictors and change variables are found in Table 3, which shows that most associations were non-significant. Several relationships between potential predictors and quality of life were statistically significant, however.

**Table 3:** Associations between sociodemographic, clinical, self-concept variables, and outcome variables (change) after completing BEL and at six-month follow-up

Variables	Occ.eng.^a^	Occupational balance						QOL
		Activity level	Work	Leisure	Home	Self-care	General balance	
Age								
BEL end	NS	*p* = .020 r_s_ = −.234	NS	NS	NS	NS	NS	*p* = .031 r_s_ = .217
Follow-up	NS	*p* = .013 r_s_ = −.257	NS	NS	NS	NS	NS	NS
Sex								
BEL end	*p* = .069	NS	NS	NS	NS	*p* = .082	NS	NS
Follow-up	NS	NS	NS	NS	NS	NS	NS	NS
Marital status								
BEL end	NS	NS	NS	NS	NS	NS	NS	NS
Follow-up	NS	NS	*p* = .026	NS	NS	NS	NS	NS
Living situation								
BEL end	NS	NS	NS	NS	NS	NS	NS	NS
Follow-up	NS	NS	NS	NS	NS	NS	NS	NS
Education								
BEL end	NS	NS	NS	NS	NS	NS	NS	NS
Follow-up	NS	NS	NS	NS	NS	NS	*p* = .089	NS
Having children								
BEL end	*p* = .010	*p* = .035	NS	NS	NS	NS	NS	NS
Follow-up	NS	*p* = .005	NS	NS	NS	NS	NS	NS
Having friend								
BEL end	NS	NS	NS	*p* = .001	NS	NS	NS	*p* = .012
Follow-up	NS	NS	NS	*p* = .004	NS	NS	*p* = .007	NS
Having seen friend								
BEL end	NS	NS	NS	*p* = .016	NS	NS	NS	*p* = .037
Follow-up	NS	NS	NS	NS	*p* = .043	NS	NS	NS
Self-esteem								
BEL end	NS	NS	NS	NS	*p* = .072 r_s_ = −.182	NS	NS	*p* = .074 r_s_ = .180
Follow-up	NS	NS	NS	NS	NS	NS	NS	*p* = .012 r_s_ = .264
Self-mastery								
BEL end	NS	NS	NS	*p* = .020 r_s_ = −.234	NS	NS	NS	NS
Follow-up	NS	NS	NS	NS	NS	NS	NS	NS
GAF-S ^b^								
BEL end	NS	NS	NS	NS	NS	NS	NS	*p* = .019 r_s_ = .235
Follow-up	NS	NS	NS	NS	NS	NS	NS	*p* = .016 r_s_ = .253
GAF-F ^c^								
BEL end	NS	NS	NS	NS	NS	NS	*p* = .096r_s_ = −.170	*p* = .009 r_s_ = .261
Follow-up	NS	NS	NS	NS	NS	NS	*p* = .007 r_s_ = −.231	*p* = .029 r_s_ = .231
Mood disorder/other								
BEL end	*p* = .059	NS	NS	NS	NS	NS	NS	*p* = .062
Follow-up	NS	NS	NS	NS	NS	NS	NS	NS
Setting^d^								
BEL end	NS	NS	NS	NS	NS	NS	NS	*p* = .081
Follow-up	NS	NS	NS	NS	NS	NS	NS	NS

^a^Occupational engagement. ^b^Symptom severity. ^c^Level of functioning. ^d^Out-patient psychiatry or community-based day centres. Internal attrition in a number of subjects occurred on the variables, between 3 and 52 and at Time = 1 and between 40 and 42 at Time = 2

Regarding the categorical variables in Table 3, only *p*-values for differences between categories are shown. Table 4 therefore presents mean change on outcomes for the groups forming those categorical variables, and for which statistically significant differences were found, thus indicating which groups had the higher and lower values, respectively.

**Table 4:** Mean values for change on outcome variables (at BEL end and the six-month follow-up), split on the categorical variables showing statistically significant associations with outcomes according to Table 3

Categorical variables	Occ.eng.^a^	Occupational balance						QOL
		Activity level	Work	Leisure	Home	Self-care	General balance	
Women/Men								
BEL end	3.2/1.1					0.3/−0.1		
Follow-up								
Married/single								
BEL end								
Follow-up			−0.3/0.3					
Children/no children								
BEL end	4.1/1.3	0.3/0.4						
Follow-up		0.2/0.5						
Having friend/no friend								
BEL end				−0.01/1.0				6.9/6.2
Follow-up				0.12/0.27			1.0/0.7	
Seen/not seen friend								
BEL end				−0.1/0.5				19.1/15.2
Follow-up					0.3/−0.1			
Mood or anxiety/other								
BEL end	1.4/3.8							16.4/19.0
Follow-up								
Setting ^b^								
BEL end								17.1/19.9
Follow-up								

^a^ Occupational engagement. ^b^ Outpatient psychiatry or community-based day centres

### Multivariate analyses

#### Baseline predictors of clinically important improvement after completed BEL intervention

##### Occupational engagement after completed BEL

The change variable based on occupational engagement was associated with being a woman, having children, and a diagnosis other than depression and/or anxiety (Tables 3 and 4). According to the regression analysis the only indicator of belonging to the group with clinically important improvement on occupational engagement after completed BEL was having children (OR 3.94, *p* = 0.020, CI 1.240–12.548) (Table 5). The OR indicates that the group with children had an almost fourfold chance of belonging to the group with improved occupational engagement. The model correctly classified 67% of the cases and explained 18% of the variance in occupational engagement (Nagelkerke R Square). Moreover, the model was supported by a non-significant Hosmer and Lemeshow test (*p* = 0.843).

**Table 5:** Predictors of clinically important change based on multi-variate analyses

Predictors	Occ. eng.^a^	Occupational balance						QOL
		Activity level	Work	Leisure	Home	Self-care	General balance	
Female gender								
BEL end						OR 5.96 *p* = .022 CI 1.298–27.357		
Follow-up								
Having children								
BEL end	OR 3.94 *p* = .020 CI 1.240–12.548							
Follow-up		OR .268 *p* = .018, CI 0.090–0.802						
Having a close friend								
BEL end				OR 4.3 *p* = .023 CI 1.218–15.091				
Follow-up				OR 5.29 *p* = .005 CI 1.651–16.971				
Higher self-esteem								
BEL end					OR 0.412 *p* = 0.01 CI 0.197–0.858			
Follow-up								
Higher psycho-social function-ing								
BEL end								
Follow-up							OR 0.95 *p* = .027 CI 0.902–0.994	
Higher education level								
BEL end								
Follow-up							OR 0.30 *p* = .039 CI 0.093–0.939	

^a^ Occupational engagement

##### Activity level after completed BEL

The change variable based regarding activity level was associated with not having children and younger age (< 40) (Tables 3 and 4). None of these could explain, however, clinically important improvement on activity level after completed BEL; the *p*-values were 0.473 and 0.629 respectively.

##### Occupational balance after completed BEL

The change variable based on occupational balance in the work domain showed no associations with the targeted predictor variables (Table 3).

Change on occupational balance in the leisure domain was associated with self-mastery, having a close friend and having seen a friend during the last week (Tables 3 and 4). According to the regression analysis, the only indicator of belonging to the group with improved balance in the *leisure domain* was having a close friend (OR 4.3, *p* = 0.023, CI 1.218–15.091) (Table 5). The OR indicates that the group who had a close friend had a more than fourfold chance of belonging to the group with clinically important improvement in the *leisure domain* of occupational balance, compared to those who did not have a close friend. The model correctly classified 73% of the cases and explained 16% of the variance in the dependent variable (Nagelkerke R Square). Moreover, the model was supported by a non-significant Hosmer and Lemeshow test (*p* = 0.944).

The change variable based on occupational balance in the *home chores domain* was associated with self-esteem (Table 3). The regression analysis with only this independent variable showed that for each step of increased self-esteem, the likelihood of belonging to the group with improved balance in the *home chores domain* after completed BEL was reduced to 40% (OR 0.412, *p* = 0.018, CI 0.197–0.858) (Table 5) of the chances for those who had one step lower a score. The model correctly classified 68% of the cases and explained 8.4% of the variance in occupational balance in the *home chores domain*. As there was only one predictor variable in the regression model, the Hosmer-Lemeshow test was not applicable.

Change on occupational balance in the *self-care domain* was associated with being a woman (Tables 3 and 4). The regression model showed that being a woman was an indicator of belonging to the group with better occupational balance in the *self-care domain* (OR 5.96, *p* = 0.022, CI 1.298–27.357) (Table 5). The OR indicates that women had an almost six-fold chance of belonging to the group with improved balance in the *self-care domain*. The model correctly classified 71% of the cases and explained 11% of the variance. With only one predictor variable entered in the model, the Hosmer-Lemeshow test was not applicable.

Improved *general occupational balance* was associated with psychosocial functioning. This lone independent variable in the regression model could not explain the variance in *general occupational balance*, as indicated by *p* = 0.079.

##### QOL after completed BEL

Change on QOL was associated with higher age (> 40), having a close friend and having seen a friend within the past week. Furthermore it was associated with better psychosocial functioning and less psychiatric symptoms, a diagnosis other than depression and/or anxiety, better self-esteem, and having received the intervention in the community mental health services (vs. specialized psychiatric services) (Tables 3 and 4). None of these independent variables could explain clinically important improvement on QOL in the regression model; the *p*-values ranging between 0.369 and 0.998.

### Baseline predictors of clinically important improvement at a six-month follow-up after completed BEL

#### Occupational engagement at the six-month follow-up

None of the targeted predictor variables showed any association with change in occupational engagement at the six-month follow-up (Table 3) and no regression analysis was thus performed.

#### Activity level at the six-month follow-up

Change on activity level was associated with younger age (< 40) and having children (Tables 3 and 4). According to the regression analysis, the only indicator of belonging to the group with clinically important improvement on activity level was having children (OR 0.268, *p* = 0.018, CI 0.090–0.802) (Table 5). The OR indicates that the chances for the group with children of belonging to the group with improved activity level was 27% of the chances of those who did not have children. The model explained 12% of the variance in activity level, classified 66% of the cases correctly, and was supported by a non-significant Hosmer and Lemeshow test (*p* = 0.451).

#### Occupational balance at the six-month follow-up

Change on occupational balance in the *work domain* at the follow-up was associated with being single (vs. being married or co-habiting) (Tables 3 and 4). This independent variable could not explain clinically important improvement in occupational balance in the *work domain* in the regression analysis, as indicated by *p* = 0.283.

The change variable based on occupational balance in the *leisure domain* at follow-up was associated with having a close friend (Tables 3 and 4). The regression model indicated that the group that had a close friend had a more than fivefold chance of belonging to the group with clinically important improvement on occupational balance in the *leisure domain,* compared to those who had no such friend (OR 5.29, *p* = 0.005, CI 1.651–16.971) (Table 5). The model correctly classified 75% of the cases and explained 12% of the variance. With only one predictor variable in the model, the Hosmer-Lemeshow test was not applicable.

The change variable pertaining to occupational balance in the *home chores domain* was associated with having seen a friend during the last week (vs. not having seen a friend) (Tables 3 and 4). This independent variable could not explain clinically important improvement in the *home chores domain* at the six-month follow-up, however, as indicated by *p* = 0.061 in the regression analysis.

Change on occupational balance in the *self-care domain* at the six-month follow-up showed no associations with the targeted predictor variables (Table 3) and no regression analysis was performed.

Change in general occupational balance was associated with having a close friend, education level and psychosocial functioning (Tables 3 and 4). According to the regression analysis, the strongest indicator for clinically important improvement on general occupational balance was psychosocial functioning (OR 0.95, *p* = 0.027, CI 0.902–0.994) (Table 5). The OR indicates that for each increased scale step in psychosocial functioning the chances of belonging to the group with clinically important improvement on general occupational balance were reduced to 95% compared to those who had one point lower a score. Furthermore, for those with an education at college or university level, the chance of belonging to the group with clinically important improvement on general occupational balance was 30% of that for the participants with lower levels of education (Table 1), (OR 0.30, *p* = 0.039, CI 0.093–0.939) (Table 5). The model correctly classified 71% of the cases and explained 23% of the variance in general occupational balance and was supported by a non-significant Hosmer and Lemeshow test (*p* = 0.491).

#### QOL at the six-month follow-up

QOL change was associated with self-esteem, psychosocial functioning and psychiatric symptoms (Tables 3 and 4). These were the independent variables in the regression analysis addressing clinically important improvement on QOL at the six-month follow-up. None of them became significant, *p-*values ranging between 0.913 and 0.986.

## Discussion

This study explored whether care context or socio-demographic, clinical and self-factors could predict clinically important improvements in the outcomes of occupational engagement, activity level, occupational balance, and QOL among BEL participants. Bivariate associations between potential predictors and changes in outcomes were first performed to identify which predictors to enter in regression analyses addressing clinically important improvements. Improvements at completed BEL and at a follow-up 6 months later were in focus. Overall, few factors were found that predicted clinically important improvement in the targeted outcomes. This can be viewed as a positive finding, as it means that making changes in occupational aspects and QOL were available to the diverse group that made up the BEL participants, often regardless of their socio-demographic details, diagnosis, psychosocial functioning level, severity of symptoms, or their self-esteem or self-mastery scores at baseline. Furthermore, as care context was not found to be associated with change in the bivariate analyses (except for a weak association with QOL upon completion of BEL), nor to be a predictor of improvement in any of the regression models, BEL seems to be a suitable intervention in both out-patient psychiatry as well as community-based mental health settings.

### Socio-demographic factors as predictors

Of all factors studied, the selected socio-demographic factors were the strongest predictors of belonging to the groups that made clinical improvements through the BEL intervention, as well as 6 months after completion. Having a close friend was found to be one of the strongest predictors. People who reported that they did not have a close friend had a decreased chance of belonging to the improved group in the occupational balance leisure domain at BEL completion, as well as at the six-month follow-up. This group was relatively small (cf. Table 1), but the findings suggest that working towards shaping friendships could preferably be incorporated in the BEL intervention. Interestingly, the findings from qualitative studies with BEL participants suggested that meeting others and making friends was indeed an important aspect of the intervention [16, 51]. Furthermore, having accountability to someone helped participants make progress towards their goals [52], which corresponds well with what has been proposed to support personal recovery [8, 53]. Furthermore, new research suggests that engaging in occupations that involve connecting with others and are categorized as fun (versus meaningful or obligatory) can activate the reward pathways of the brain which can affect positive mood [54]. These results along with the current study’s findings can inform future studies on the benefits and interactions of social connection, occupational balance and leisure activities.

Having a friend is related to one’s social network [55] and to social support, which have been suggested as important factors to focus on in interventions for mental health service users [30, 56], as well as recovery-oriented mental health care [57]. In addition to the occupational balance leisure domain, having a close friend was also found to be correlated with QOL at BEL end and with general occupational balance at the six-month follow-up. Related to this, having seen a friend during the last week was also found to be associated with QOL and the occupational balance leisure domain at completed BEL, as well as home chores at the six-month follow-up. However, none of these were found to be predictors of clinically important improvement in the outcome variables according to the multivariate analysis. Having a friend has been found in previous research to be associated with QOL [58], though not a predictor [32], which was supported by the current study. However, other studies have found that having supportive social interactions could be a predictor of QOL [59, 60] and improved QOL [59]. Having a close friend has been shown to be associated with occupational engagement in previous research [19]. The current study did not find a correlation between the two, which could be explained by the fact that this study focused on factors that would predict change. It is possible that those who had a close friend, which were most participants in this study, already had better scores of occupational engagement to begin with, and thus, less room for improvement.

Interestingly, having children was found to increase chances approximately four-fold for belonging to the improved group in occupational engagement at BEL completion, while having children also decreased the chances for belonging to the improved group on activity level at follow-up. These findings should not be seen as contradictory, since occupational engagement targets the individual’s experience of meaning and sense of agency in occupation rather than the actual amount of performed activities (activity level). Thus, those who had children seem to have been able to increase their level of engagement in activities without increasing the number of activities in which they were involved. On the other hand, those without children may have had more time to devote to exploring other activities, and thus increase their activity level. This would be in line with Eklund and Argentzell [27] who found that mental health service users with children tended to be over-occupied, in contrast with those who did not have children. Results from a qualitative study from the larger BEL project adds to this reasoning. Some BEL participants reported that not having enough time to work on one’s personal goals due to family or other life demands was a hindering factor that affected their participation in BEL and working towards change [52].

Being a woman was found to be associated at BEL end with changes in terms of improved occupational engagement and general occupational balance. Female gender was also associated with the occupational balance self-care domain and was a predictor that increased the chances six-fold of belonging to the group with a clinically important change in balance within the self-care domain. Gender was not found to be a predictor for any other change variables, including all outcome variables at the six-month follow-up. A study on occupational balance found that those with children, which were more women than men, tended to be over-occupied in the home chores domain and under-occupied in self-care [27]. Other studies have found that women more often experience imbalance in their daily lives due to high demands at home, resulting in decreased self-care, rest and recovery, which can affect their health [61–63]. Another study found that, compared with women without a mental illness, women with personality disorders experienced higher stress, less subjective balance, and were found to value rest less [64]. Thus, in relation to the current study’s findings, it is possible that female mental health service users had more room for improvement in the area of self-care. For both genders, setting boundaries, prioritizing self, and caring for a valued self were important parts of the process of making changes through the BEL intervention, per participant interviews [52]. Similar strategies might lie behind the current study’s findings and give an explanation of factors that helped create more time for self-care.

Education level was another sociodemographic variable that was found to be a predictor for general occupational balance, as those with a higher education had a decreased chance of being in the clinically important change group 6 months after BEL completion. This supports other research that has found education to be associated with change after an activity-based lifestyle intervention [65]. In that study, however, which focused on predictors of outcomes of an intervention for women with stress-related disorders, higher education was beneficial for change. This highlights one of several possible differences between activity-based lifestyle interventions and that such interventions need to be tailored towards the needs of specific groups.

### Clinical and self-factors as predictors

This study found that only a few of the studied clinical and self-factors could predict belonging to the groups with a clinically important improvement. Those with better psychosocial functioning at baseline had a decreased chance of improved general occupational balance at the six-month follow-up. This result appears logical as lower psychosocial functioning may give more room for improvement in functioning, in turn entailing improved occupational balance. Better psychosocial functioning, however, together with less symptoms, was associated with improved QOL at BEL end and the six-month follow-up. This supports previous research that less severe symptoms and improved clinical factors support QOL [56, 58, 59, 66–68]. Nevertheless, none of these potential predictors became significant in the regression models addressing clinically important improvement in QOL.

Self-mastery was found to have a negative association with change on occupational balance in the leisure domain at BEL end, though no association was found at the six-month follow-up. Self-mastery has been found previously to be associated with occupational engagement [20], though not in the current study. Participants with better self-esteem had a decreased chance of belonging to the improved group regarding occupational balance in the home chores domain at BEL completion. This is not in line with other studies that suggest that self-esteem tends to be positively associated with occupational balance [12, 27]. Again, however, it could be that those with lower scores on self-esteem in the present study had more room for improvements, which may in turn have positively affected their perception of occupational balance in the home chores domain. Thus, one outcome may have reinforced the other, but investigating if that was the case was not in line with the aim of this study. Furthermore self-esteem was associated with QOL changes at BEL completion and 6 months following, though was not found to predict clinically important improvement in QOL at either of these points. Self-esteem and self-mastery have also been found to be strong determinants of subjective QOL [34, 58]. These previous studies were cross-sectional, however, and did not focus on change or intervention outcomes. Another study found that an intervention that focused on and increased self-esteem also improved QOL, health, and social relationships [69]. The current study suggests that, according to bivariate analyses, self-factors at baseline were associated with change on occupational balance, activity level and QOL following the BEL intervention, though not strong predictors of making clinically important improvements.

## Methodological discussion

The BEL intervention included 106 participants from specialized psychiatry and 27 participants from community-based psychiatry. Thus, there was a skewness in recruitment from the two care contexts, which was unintentional. This difference mirrors, however, how the psychiatric services with access to an occupational therapist are organized in Sweden, with fewer opportunities in community-based psychiatry. Nevertheless, psychiatric care context was only weakly associated with QOL upon completion of BEL, and did not become a predictor in the multivariate analysis.

All eligible service users at the time of the project were invited to the study. Due to the use of gatekeepers for recruiting participants and recording those who opted in or not, as well as multiple research assistants over the length of the study, there were some failures in the communication and the number of nonparticipants could not be exactly estimated making the generalisability of this study an issue. Another issue was dropout from the intervention, which included 33 participants (25%) from baseline to completed BEL. However, those who dropped out did not differ from the completers on sociodemographic or clinical characteristics. Efforts to prevent attrition included pre-intervention interviews to ensure that the project aims aligned with participants’ personal aims. Group leaders also contacted participants after a missed session to update them on the content and discussions. Reasons for dropping out included health or family issues and increasing time in employment.

Regarding the statistical analyses the authors chose to dichotomize the targeted outcome variables at a cut-off value of change/improvement at an effect size of 0.5, which has been suggested to be of clinical importance. Dichotomization of variables has been argued to cause a decrease in measured strength of associations [50], due to loss of some of the variance in the dichotomized variable. This needs to be taken into consideration in the present study and would explain why only a few of the associations based on bivariate analyses (where the full variation in the target variables was kept) became statistically significant in the multivariate analyses (where the dependent variable was dichotomized). The choice of method for estimating clinically important change may also be discussed, but Cohen’s ES used in this study has been shown to be reliable compared to other mathematical methods [70]. Nevertheless, an estimate based on patients’ appraisal of what would be an important change [71] would be a stronger indicator. This was, however, not feasible within the scope of the current study. Regarding the predictors, they generally explained a small portion of the variance in the targeted outcome variables. This together with the fact that some of the confidence intervals were wide indicates that the results should be interpreted with caution.

Another issue was internal attrition, which is common in studies where self-report questionnaires are used, as in the present study. One variable, occupational engagement (POES instrument), had an attrition of 52, which was due to the fact that participants filled it out as part of the BEL intervention, and group leaders were instructed to make copies and send to the research team. Failure in communication and follow-up made it so that some of these instruments were lost.

The occupational balance ratings indicated that the majority of the participants in the present study were under-occupied and few were over-occupied, thus a positive change showed progress towards better occupational balance. Moreover, this study focused explicitly on investigating sociodemographic and self-factors as predictors of the targeted outcome variables and the baseline values of the targeted outcome variables were not controlled for in the multivariate analyses. The rationale behind this strategy was to inform decisions in clinical practice, where sociodemographic and clinical factors are often known and can make a basis for how to compose a BEL group, whereas self-factors are more seldom routinely assessed in the occupational therapy arenas where the BEL intervention is delivered. Furthermore, regarding sociodemographic predictors women were over-represented in the study, as were the participants who stated that they had a close friend; both of these circumstances may have had an unknown impact on the results.

## Conclusions

Overall, few socio-demographic, self-related, or clinical factors were found to predict change in occupational engagement, activity level, occupational balance, or QOL. This study, together with previous studies showing positive results, suggests that BEL can be an appropriate intervention in both community and clinical settings, and can support clinically important improvements in occupational aspects and QOL for participants with diverse socio-demographic, clinical, and self-related characteristics. Having a close friend, having children or not, and being a woman were found to be the strongest predictors of improvement in different areas pertaining to occupational engagement, activity level, and occupational balance. Future research could explore why BEL attracted more female participants, as well as delve deeper into factors that promote occupational balance for women and men with mental illness in the self-care domain. Investigating whether the connection between higher education and less change in general balance can be replicated among BEL participants is another topic for future research. This study suggests that “having a close friend” is a particularly important aspect of socio-demographic information to gather in research studies of activity-based lifestyle interventions.

## Data Availability

The Swedish Act regarding the Ethical Review of Research Involving Humans restricts data sets from being publicly available. The data sets used in this study are available from the corresponding author upon request.

## Abbreviations

BEL: Balancing Everyday Life
C: Cut value
CI: Confidence interval
ES: Effect size
GAF: Global Assessment of Functioning
GAF-F: GAF Functioning
GAF-S: GAF – Symptoms
ICD: International Classification of Diseases
MANSA: Manchester Short Assessment of Quality of Life
OR: Odds ratio
POES: Profiles of Occupational Engagement among people with Severe mental illness
QOL: Quality of Life
RCT: Randomized Controlled Trial
SD_0_: Standard deviation at baseline
SDO-OB: Satisfaction with Daily Occupations and Occupational Balance
